# Preclinical development of an mRNA-based multiepitope immunotherapeutic for glioblastoma

**DOI:** 10.1007/s00262-025-04178-x

**Published:** 2025-10-06

**Authors:** Johannes Lutz, Randi K. Feist, Tim Sonntag, Esteban Peguero-Sánchez, Katharina Wolter, Ronja Bick, Jens Bauer, Juliane S. Walz, Regina Heidenreich

**Affiliations:** 1 CureVac SE, Friedrich-Miescher-Strasse 15, 72076 Tübingen, Germany; 2 Department of Peptide-Based Immunotherapy, Institute of Immunology, University and University Hospital Tübingen, Tübingen, Germany; 3 Cluster of Excellence iFIT (EXC2180) “Image-Guided and Functionally Instructed Tumor Therapies”, University of Tübingen, Tübingen, Germany; 4 German Cancer Consortium (DKTK), Partner Site Tübingen, DKFZ and University Hospital Tübingen Partnership, Tübingen, Germany; 5 Clinical Collaboration Unit Translational Immunology, Department of Internal Medicine, University Hospital Tübingen, Tübingen, Germany

**Keywords:** Glioblastoma, Immunotherapy, mRNA-based immunotherapeutic, Cancer vaccine

## Abstract

Glioblastoma (GBM), an aggressive brain tumour associated with poor prognosis and high recurrence rate, has limited clinical treatment options. However, novel immunotherapeutics targeting over-presented epitopes of tumour-associated antigens (TAAs) represent a promising solution. Here we describe the preclinical development of CVGBM, an mRNA-based immunotherapeutic candidate for GBM consisting of a nucleotide-unmodified mRNA encapsulated in lipid nanoparticles (LNP). CVGBM mRNA encodes a fusion protein comprising eight TAA-derived epitopes that have previously induced T-cell responses in patients with GBM as peptide-based immunotherapeutics: five restricted to class I human leukocyte antigen (HLA) allele A*02:01 and three restricted to various class II HLA-DR alleles. Translation and processing of the mRNA-encoded fusion protein and presentation of derived epitopes on HLA molecules were confirmed in human cell lines after lipofection with CVGBM mRNA. Immunopeptidomics confirmed the presentation of four of the six HLA-A*02:01-restricted epitopes; however, HLA class II-bound epitopes were not detected. Administration of CVGBM to mice demonstrated functionality of the immunotherapeutic in vivo by inducing CD8^+^ and CD4^+^ T-cell responses. As CVGBM requires an intact immune system for its mode of action, it could not be tested in xenograft models. Instead, anti-tumoural efficacy was demonstrated for a surrogate mRNA-based immunotherapeutic, which has a similar mRNA and protein design as CVGBM but encodes a fusion protein comprising epitopes of the murine B16.F10 melanoma cell line. Administration of the surrogate immunotherapeutic prolonged median survival time of B16.F10 tumour-bearing mice relative to controls. Based on these results, a Phase I clinical trial with CVGBM was started in HLA-A*02:01-positive patients with surgically resected MGMT-unmethylated GBM (NCT05938387).

**Supplementary Information:**

The online version contains supplementary material available at 10.1007/s00262-025-04178-x.

## Introduction

Glioblastoma (GBM) is the most commonly occurring malignant tumour of the central nervous system (CNS), accounting for half of all malignant brain or CNS tumours [1]. Between 2015 and 2019, the annual age-adjusted incidence of GBM in the USA was 3.26/100,000 persons and was 1.6 times more common in males than females [1]. The disease prognosis is poor, with an estimated five-year overall survival (OS) rate of 6.9% [1] and a median OS of 12–16 months after diagnosis [2, 3].

The current standard of care for GBM includes surgical resection and post-surgery radiotherapy with or without temozolomide (TMZ) chemotherapy [4, 5]. Due to the invasiveness of tumour cells and limitations in GBM detection and removal, surgical resection can improve survival and quality of life but is almost never curative [6]. Despite standard of care treatment, GBM recurrence is inevitable and there is no agreed salvage therapy post-recurrence [4, 5]. Therefore, an urgent need for new GBM treatment approaches exists to improve patient outcomes.

Immunotherapies such as checkpoint inhibitors against cytotoxic T-lymphocyte-associated protein 4 (CTLA-4) and programmed death-1 (PD-1)/PD ligand 1 (PD-L1) have represented a major advancement in the treatment of several types of solid tumour; however, these treatments have shown only very limited benefits in patients with GBM [7–9]. Treatment of GBM is also hampered by its high degree of intratumoural heterogeneity, which makes the identification of suitable antigens for immunotherapy difficult. Tumour cell heterogeneity and an immunosuppressive microenvironment contribute to primary GBM immunoresistance, which dampens anti-tumour responses by macrophages and T cells [7, 10]. GBM immunoresistance may also be acquired in response to therapy, through changes in checkpoint expression, epigenetic changes, and loss of tumour antigen expression [10]. Approaches that overcome these barriers are needed and various other immunotherapies, including antigen-based immunotherapies (cancer vaccines), have been explored as treatment options for GBM.

Antigen-based immunotherapies are designed to induce cytotoxic T-cell responses against antigens expressed by the tumour, for example, tumour-associated antigens (TAAs) that are over-expressed in tumours compared with healthy tissue, resulting in tumour cell death [8, 11]. Various modalities to deliver these antigens, for example, as peptides or antigen-loaded dendritic cells, have been explored in clinical studies of patients with GBM [12–15]. Two examples are the Phase I GAPVAC-101 study [14] and the Phase I/II IMA950 study [15], which tested peptide-based immunotherapeutics containing epitopes from unmutated TAAs that are either over-expressed or over-presented in GBM. Both immunotherapeutics, which shared some peptides, were well tolerated and induced epitope-specific CD8^+^ T-cell responses [14, 15]. While these studies demonstrated that antigen-based immunotherapeutics can elicit T-cell responses in patients with GBM, evidence of robust and durable efficacy is limited despite the apparently prolonged overall survival in the GAPVAC-101 trial compared with historical controls. Therefore, we decided to test whether an antigen-based messenger ribonucleic acid (mRNA) therapeutic could overcome this limitation.

mRNA therapeutics have recently become a key player in cancer immunotherapeutic development owing to their versatile design and ability to encode various antigens [16, 17]. Clinical trials of mRNA-based immunotherapeutics encoding unmutated full-length TAAs in checkpoint inhibitor-treated melanoma [18] and individualised mRNA-based immunotherapeutics encoding patient-specific mutated tumour neoantigens in pancreatic and lung cancer [19, 20] have shown encouraging results. mRNA-based immunotherapeutics have the potential to be especially effective in immunologically “cold” tumour types such as GBM [17, 21].

To develop a broadly applicable off-the-shelf mRNA-based immunotherapeutic for GBM, we designed CVGBM, which is based on CureVac’s platform of chemically unmodified mRNA encapsulated in lipid nanoparticles (LNP) [22]. CVGBM encodes eight unmutated TAA-derived epitopes that have been selected to cover a large proportion of patients with GBM and are concatenated into a fusion protein for optimal epitope presentation. These epitopes comprise five human leukocyte antigen (HLA)-A*02:01-restricted class I and three HLA-DR-restricted class II T-cell epitopes, as well as a class I reporter epitope. The epitopes encoded by CVGBM have previously induced T-cell responses in the GAPVAC-101 or IMA950 studies in patients with GBM when given as peptide-based immunotherapeutics in combination with adjuvants [14, 15]. Here, we describe the preclinical development and characterisation of CVGBM.

## Materials and methods

### mRNA-based immunotherapeutics

All tested mRNA-based immunotherapeutics comprised unmodified nucleotides and included the same non-coding mRNA elements as CV2CoV, a vaccine candidate against SARS-CoV-2 based on CureVac’s RNActive® mRNA technology platform [23]. For in vivo use, all mRNAs were delivered using LNP technology of Acuitas Therapeutics (Vancouver, Canada), consisting of ionisable aminolipid, phospholipid, cholesterol, and a PEGylated lipid.

### TAA expression in the target population

To estimate the coverage of the patient population with GBM for CVGBM, TAA expression was simulated for 100 clinical trials of 50 patients each, using gene expression data from 155 patients with GBM obtained from The Cancer Genome Atlas database [24]. A gene expression of ten transcripts per million (TPM) was used as the threshold to consider a gene expressed in an individual patient’s tumour.

### Detection of reporter epitopes on murine bone marrow-derived dendritic cells

Bone marrow cells were harvested from C57BL/6 mice and cultured at 1 × 10^6^ cells/ml with complete RPMI medium (Gibco) containing granulocyte–macrophage colony-stimulating factor (GM-CSF) (20 ng/ml, Miltenyi) and interleukin (IL)-4 (5 ng/ml, Miltenyi). After 3 days, the same volume of complete RPMI medium with GM-CSF and IL-4 was added. At Day 6, cells had differentiated into bone marrow-derived dendritic cells (BMDCs). 3 × 10^6^ BMDCs were mixed with 20 µg mRNA (230 µl total) in Opti-MEM medium (Gibco) and electroporated using a Gene Pulser Xcell electroporation system (Bio-Rad; Square wave, 300V, 1 pulse of 6 ms, 2 mm gap). After electroporation, cells were transferred to RPMI medium with a final concentration of 0.1 µg/ml lipopolysaccharides (Sigma-Aldrich). As positive controls, untransfected cells were pulsed with SIINFEKL or Eα peptides (10 µg/ml). Cells were harvested, incubated with LIVE/DEAD™ Fixable Aqua stain (Invitrogen) and Fc-block (Invitrogen), and stained using T-cell receptor (TCR)-like antibodies recognising the SIINFEKL epitope presented on major histocompatibility complex (MHC) class I molecule H-2Kb (clone 25-D1.16, BioLegend), or the Eα epitope presented on MHC class II molecule I-Ab (clone Y-Ae, Santa Cruz). Alternatively, cells were treated with Cytofix/Cytoperm solution (BD Biosciences) and Fc-block and stained intracellularly with an antibody recognising the haemagglutinin (HA) 0.11 epitope tag (BioLegend). All cells were analysed on a flow cytometer.

### Detection of hepatitis B virus reporter epitope on human cells

HEK293T cells were transfected with 5 µg of lipofectamine-formulated CVGBM mRNA or mock transfected with the same amount of lipofectamine 2000 (Invitrogen; mRNA: lipofectamine = 1 µg: 2 µl). Cells were stained with a TCR-like antibody recognising the hepatitis B virus (HBV)-001 epitope presented on HLA-A*02:01 molecules (clone c18/A2; Creative Biolabs; 1 µg/ml), an AF647-labelled goat anti-mouse immunoglobulin G secondary antibody (Invitrogen), and analysed on a flow cytometer.

### Immunopeptidomic analysis of CVGBM-transfected human cells

HEK293T or THP-1 cells were seeded in cell culture dishes (Ø = 15 cm; 6.4 × 10^6^ HEK293T or 8 × 10^6^ THP-1 cells per dish) and lipofected the next day with 32 µg mRNA per dish of CVGBM mRNA or a control mRNA encoding an unrelated protein (lipofectamine 2000; Invitrogen; mRNA: lipofectamine = 1 µg: 2 µl). After 6 h, cells were harvested by pipetting or scraping, washed twice in ice-cold phosphate buffered solution, and snap-frozen at − 80 °C as dry pellets.

HLA class I and II molecules were isolated by standard immunoaffinity purification [25] using the pan-HLA class I-specific mAb W6/32, the pan-HLA class II-specific mAb Tü39, and the HLA-DR-specific mAb L243 (all produced in-house) to extract HLA ligands.

Liquid chromatography-coupled tandem mass spectrometry (LC–MS/MS) analysis and data processing were performed as described previously [26]. In brief, peptide samples were separated by nanoflow high-performance liquid chromatography (RSLCnano, Thermo Fisher Scientific) and analysed in five technical replicates for each sample. Eluting peptides were analysed in an online-coupled LTQ Orbitrap Fusion Lumos mass spectrometer (Thermo Fisher Scientific) using a top speed collision-induced dissociation fragmentation method. The SEQUEST HT search engine (University of Washington) was used to search the human proteome (Swiss-Prot database, 20,279 reviewed protein sequences, September 27 2013, with the CVGBM and control sequences added) without enzymatic restriction. Precursor mass tolerance was set to 5 ppm and fragment mass tolerance to 0.02 Da. Oxidised methionine was allowed as a dynamic modification. The false discovery rate was estimated using the Percolator algorithm and limited to 5% for HLA class I and 1% for HLA class II. Peptide lengths were limited to 8–12 amino acids (aa) for HLA class I and to 8–25 aa for HLA class II. HLA class I annotation was performed using NetMHCpan 4.1 and SYFPEITHI annotating peptides with scores, or percentile rank below 2% or higher than 60, respectively.

Relative quantification of HLA ligands and volcano plot analysis was performed as described previously [27]. Briefly, the ratios of the mean areas of the individual peptides in the five label-free quantification mass spectrometry runs of each sample were calculated and unpaired, heteroskedastic two-tailed *t*-tests implementing Benjamini–Hochberg correction were performed using an in-house R script (v3.2.3). IEDB (www.iedb.org) was accessed on 17 October 2024, host: human.

### In vivo immunogenicity of CVGBM in mice

Naïve female CB6F1 mice (C57BL/6 and BALB/c F1 hybrid, 7–9-weeks old, *n* = 8 per group) were injected intramuscularly (IM) on Days 0, 6, and 13 with 5 µg mRNA per mouse of CVGBM or LNP-formulated control mRNA. On Day 20, mice were sacrificed. Splenocytes were isolated and stimulated with peptide pools covering each of the encoded GBM or HBV segments (overlapping 15-17mer peptides, shifted by 4 aa, 5 µg/ml for each peptide) or dimethyl sulfoxide (DMSO) as control in the presence of antibodies activating CD107a (BioLegend) and CD28 (BD Biosciences) for 6–7 h. After 1 h, GolgiPlug (BD Biosciences) was added. After stimulation, splenocytes were incubated with LIVE/DEAD™ Fixable Aqua stain and Fc-block (both Invitrogen), stained with fluorophore-conjugated antibodies recognising surface markers (including CD4 [BD Biosciences], Thy1.2 [BioLegend]; CD8 [Invitrogen]), incubated with Cytofix/Cytoperm (BD Biosciences), stained with antibodies recognising intracellular markers (tumour necrosis factor [Invitrogen], interferon-gamma [BD Biosciences]), and analysed on a flow cytometer.

### In vivo immunogenicity of B16 immunotherapeutic in mice

Naïve female C57BL/6 mice (8 weeks old, *n* = 5 per group) were injected IM with 5 μg mRNA-LNP on Days 0, 7, and 14. On Day 21, mice were sacrificed and splenocytes isolated. Splenocytes were analysed as described above using the indicated peptides (29 aa each, 13 aa for pan HLA-DR binding epitope [PADRE]; 5 µg/ml).

### Anti-tumour efficacy of B16 immunotherapeutic in mice

Naïve female C57BL/6 mice (7-weeks old) were challenged with 1 × 10^5^ B16.F10 tumour cells administered subcutaneously into the flank. Once tumours were palpable (Day 7), mice were injected IM with 5 µg mRNA-LNP on Days 7, 14, and 21 (*n* = 10 per group) or left untreated (*n* = 6). Tumour size was determined twice per week using a calliper. Tumour volume was calculated as: (length × width^2^ × π)/6. Tumour growth and survival were analysed for up to 100 days.

### Statistical analysis

Statistical analyses were performed using GraphPad Prism software (Version 10.3) or R. Details of each analysis are provided in the associated figure legend; *p* < 0.05 was considered statistically significant.

### Ethics

The animal experimental protocols were approved by the ethics committee of the Tübingen Regional Council. Animal studies were performed by CureVac SE or Synovo GmbH, Tübingen, Germany.

## Results

### Antigen selection and design of immunotherapeutic candidates

CVGBM was developed by selecting eight epitopes that are frequently presented on GBM tumours and have previously demonstrated immunogenicity in the GAPVAC and IMA950 trials when used as peptide-based immunotherapeutics [14, 15]. Five of these TAA-derived epitopes are restricted to the class I allele HLA-A*02:01 and three to various class II HLA-DR alleles (Table 1). The selected epitopes are derived from four proteins: brevican core protein (BCAN), neuroligin-4 (NLGN4X), receptor-type tyrosine-protein phosphatase zeta (PTPRZ1), and baculoviral inhibitor of apoptosis repeat-containing protein 5 (BIRC5; Survivin). Three of the TAAs (BCAN, PTPRZ1, BIRC5) are highly over-expressed in GBM tumours compared with healthy tissue (except testis) (Supplementary Fig. 1). NLGN4X is less over-expressed in GBM; however, the encoded epitope is over-presented on GBM tumours compared with healthy brain tissue [28].

**Table 1:** Epitopes encoded by CVGBM mRNA

Source protein	UniProt-ID	Epitope (after processing)	Peptide ID^a^	HLA	HLA class	Encoded antigen segment (29 aa)	Position of segment in source protein
BCAN	Q96GW7	ALWAWPSEL	BCA-002	A*02:01	I	EVEDE(ALWAWPSEL)SSPGPEASLPTEPAA	aa 473–501
NLGN4X	Q8N0W4	NLDTLMTYV	NLGN4X-001	A*02:01	I	HDMLPIWFTA(NLDTLMTYV)QDQNEDCLYL	aa 121–149
PTPRZ1	P23471	AIIDGVESV	PTP-003	A*02:01	I	VGTEENLDFK(AIIDGVESV)SRFGKQAALD	aa 185–213
		KVFAGIPTV	PTP-005	A*02:01	I	LTSTKSSVTG(KVFAGIPTV)ASDTFVSTDH	aa 1337–1365
		MIWEHNVEV	PTP-013	A*02:01	I	LKSTAEDFWR(MIWEHNVEV)IVMITNLVEK	aa 1804–1832
BIRC5; Survivin	O15392	TLGEFLKLDRERAKN	BIR‐002	pan-DR	II	KKQFEEL(TLGEFLKLDRERAKN)KIAKETN	aa 90–118
BCAN	Q96GW7	VKVNEAYRFRVALPAYPA	BCA-005	pan-DR	II	VARGVR(VKVNEAYRFRVALPAYPA)SLTDV	aa 92–120
PTPRZ1	P23471	EIGWSYTGALNQKN	PTP-010	pan-DR	II	RQQRKLVE(EIGWSYTGALNQKN)WGKKYPT	aa 27–55
Capsid protein; HBV	P03148	FLPSDFFPSV	HBV‐001	A*02:01	I	EFGATVELLS(FLPSDFFPSV)RDLLDTASA	aa 8–36

Each epitope (in brackets) is present in an antigen segment of 29 aa, with the epitope being flanked by aa sequences occurring in the source proteins

^a^Nomenclature as reported in Hilf et al. 2019 [14]

aa, amino acids; BCAN, brevican core protein; BIRC5, baculoviral IAP repeat-containing protein 5; HBV, Hepatitis B virus; HLA, human leukocyte antigen; IAP, inhibitor of apoptosis; NLGN4X, neuroligin-4; PTPRZ1, receptor-type tyrosine-protein phosphatase zeta

**Fig. 1:**
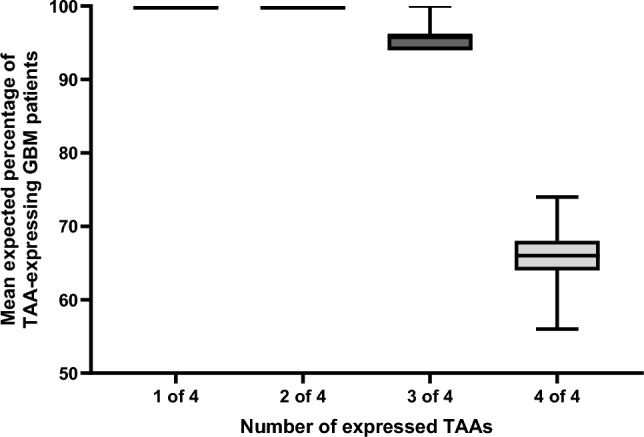
A high percentage of GBM patients are expected to express multiple antigens targeted by CVGBM. Data on the distribution of TAA expression in GBM samples (The Cancer Genome Atlas, v37.0) [24] were used to estimate the percentage of GBM patients expressing 1 to 4 of the TAAs in 100 simulated (bootstrapping) clinical trials of 50 patients each. Error bars represent maximum and minimum values. GBM, glioblastoma; TAA, tumour-associated antigen

Simulation results using gene expression data of GBM tumours from The Cancer Genome Atlas database [24] showed that a median of 96% patients express at least three of the four TAAs in their tumour (Fig. 1). Importantly, in an HLA-A*02:01^+^ patient population, epitopes of the expressed TAAs will also be presented on the tumours of most patients to the immune system, as CVGBM encodes HLA-A*02:01-restricted epitopes for three of the TAAs (BCAN, NLGN4X, PTPRZ1) and a pan-DR-restricted epitope for the fourth TAA (Survivin), which is presented by many HLA class II molecules [29].

The selected epitopes were concatenated to generate a core fusion protein encoded on a single mRNA (Supplementary Fig. 2a). Each epitope is present within an antigen segment of 29 aa, flanked by aa sequences that surround the epitope in the source proteins; allowing similar processing and presentation of the epitopes as per the full-length protein they are derived from (Table 1). The core fusion protein also contains an HLA-A*02:01-restricted epitope of HBV as a viral marker peptide, which is expected to induce more robust T-cell responses than the selected self-antigens and can therefore serve as a sensitive reporter for CVGBM immunogenicity. All segments are separated by G_4_S linkers to reduce presentation of junctional epitopes.

The core fusion protein was used to generate six different fusion protein variants with reporters (GBMr), enabling in vitro evaluation of protein translation and epitope presentation (Supplementary Fig. 2b). The GBMr fusion proteins contained localisers that targeted them to different subcellular compartments, allowing selection of the location resulting in the most favourable epitope presentation. Fusion proteins were localised to: (i) the endoplasmic reticulum (ER) and endosomal pathway using the signal peptide (SP), transmembrane and cytoplasmic domains of CTLA-4 (variants GBMr-CTLA-4 and GBMr-CTLA-4-E5); (ii) the inside of lysosomes and late endosomes using the SP and further aa sequences derived from lysosomal-associated membrane protein 1 (LAMP1; variant GBMr-LAMP1); (iii) the ER using the localisation domain of a different protein (variant GBMr-OtherLoc); or (iv) the cytosol using no localisation domains (variant GBMr-Cyto). GBMr-CTLA-4-E5 also contained a C-terminal degron consisting of five glutamic acids (E5), promoting enhanced degradation [30].

### Presentation of epitopes on MHC class I and II molecules of murine cells

Two of the reporter epitopes in the GBMr fusion proteins could be used to assess the presentation of fusion protein-derived epitopes on MHC class I and II molecules of murine cells using TCR-like antibodies: (i) the MHC class I-restricted SIINFEKL epitope derived from chicken ovalbumin and (ii) the MHC class II-restricted Eα epitope derived from mouse H2-Eα. For protein quantification, the GBMr fusion proteins also contained an HA tag (Supplementary Fig. 2b).

**Fig. 2:**
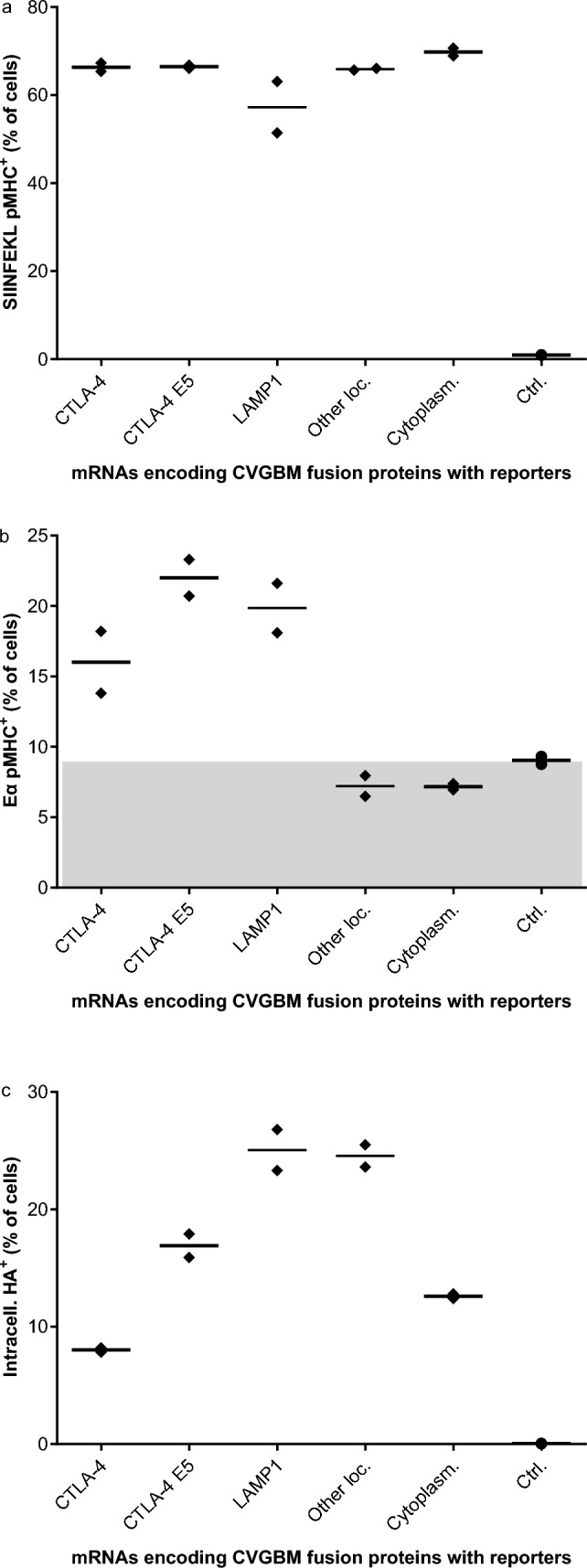
GBMr fusion protein-derived reporter epitopes are presented on MHC class I and II molecules on murine cells. BMDCs of C57BL/6 mice were electroporated with the indicated mRNA constructs and presentation of the reporter epitopes SIINFEKL on MHC class I (**a**) and Eα on MHC class II (**b**) was measured by flow cytometry 9 h post-electroporation using TCR-like antibodies. The background level of Eα pMHC staining is shown in grey. **c** Total amounts of translated fusion proteins were measured by flow cytometry using intracellular HA staining. Bars on (**a**–**c**) indicate median values of two technical replicates. BDMC, bone marrow dendritic cell; CTLA-4, cytotoxic T-lymphocyte associated protein 4; Ctrl, control; HA, haemagglutinin; LAMP1, lysosomal-associated membrane protein 1; MHC, major histocompatibility complex, pMHC, peptide major histocompatibility complex; mRNA, messenger ribonucleic acid; TCR, T-cell receptor

GBMr fusion proteins were encoded on mRNAs, which were transfected into BMDCs. Flow cytometry results demonstrated similar staining of SIINFEKL/MHC class I complexes on the surface of BMDCs 9 h after transfection with mRNAs encoding the five different GBMr proteins (Fig. 2a). In contrast, Eα/MHC class II complexes on these cells were only detected above background levels in BMDCs transfected with mRNAs encoding GBMr-CTLA-4-E5 and GBMr-LAMP1, and to a lesser extend GBMr-CTLA-4 (Fig. 2b). By 17 h post-transfection, presentation of Eα decreased to background levels for all variants, except for GBMr-CTLA-4-E5 (Supplementary Fig. 3). Steady-state protein amounts were assessed by HA staining, showing no correlation between the frequency of cells presenting the Eα epitope and the frequency of cells with detectable GBMr fusion protein (Fig. 2b and c). This is exemplified by GBMr-OtherLoc, which showed ample intracellular HA staining but no Eα presentation. Based on these data, the clinical candidate CVGBM was designed containing the core fusion protein, the CTLA-4 domains, and the E5 degron (Supplementary Fig. 2c).

### Presentation of CVGBM fusion protein-derived epitopes on human cells

**Fig. 3:**
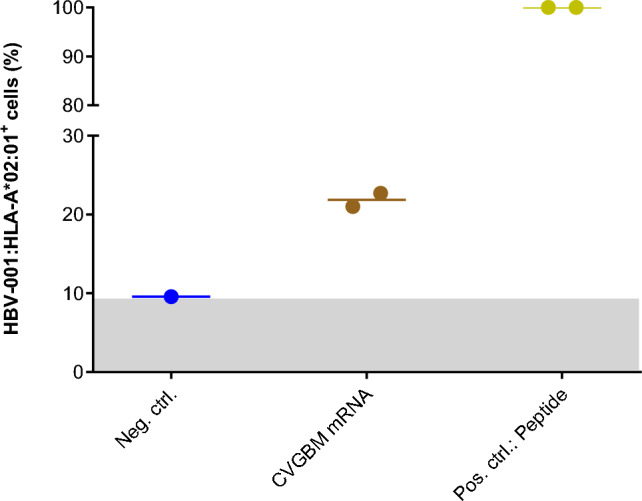
CVGBM fusion protein-derived HBV epitope is presented on HLA-A*02:01 on human cells. HEK293T cells were transfected with CVGBM mRNA in lipofectamine and presentation of HBV-001 epitope on HLA-A*02:01 was measured by flow cytometry 16 h later using a TCR-like antibody. Mock-transfected cells (blue) and cells pulsed with the HBV-001 peptide (yellow) served as controls. Bars indicate median values of two technical replicates. Ctrl, control; HBV, hepatitis B virus; neg, negative; HLA, human leukocyte antigen; mRNA, messenger ribonucleic acid; pos, positive

The presentation of CVGBM fusion protein-derived epitopes on human cells by HLA molecules was verified through two assays. First, a TCR-like antibody was used to detect the HLA-A*02:01-restricted epitope of HBV on HEK293T cells transfected with CVGBM mRNA. HEK293T cells, which endogenously express HLA-A*02:01 (Supplementary Table 1), had stronger antibody staining for HBV-001:HLA-A*02:01 complexes after transfection with CVGBM mRNA than mock-transfected cells, confirming presentation of the HBV-HLA class I epitope derived from the CVGBM fusion protein (Fig. 3).

**Fig. 4:**
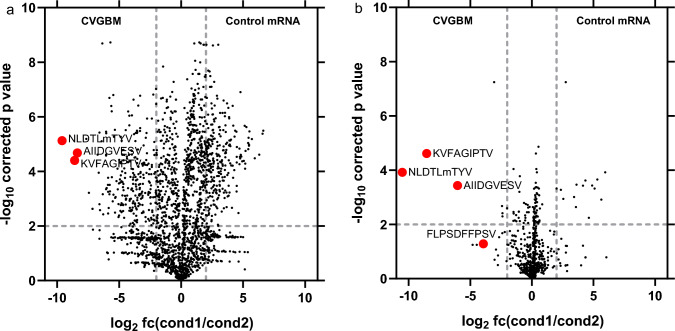
HLA class I presentation of CVGBM-derived peptides on CVGBM mRNA transfected THP-1 cells (**a**) and HEK293T cells (**b**) HLA class I epitopes were isolated by immunoprecipitation from THP-1 and HEK293T cells transfected with CVGBM mRNA (cond2) or control mRNA (cond1) and identified by untargeted LC–MS/MS (5 technical replicates). For each epitope, the fold change (fc) of the area between conditions (*X* axis) and the statistical significance of the change (*Y* axis) are plotted. Dotted lines indicate a fourfold change between conditions (vertical) and an adjusted *p*-value of 0.01 (horizontal). CVGBM-derived epitopes are depicted in larger dots and labelled with epitope identifiers. All CVGBM-derived epitopes were only detected in cells transfected with CVGBM mRNA. Therefore, a sample-specific limit of detection was calculated as the median of the five lowest detected areas and used to calculate the fold change for CVGBM-derived epitopes. cond, condition; fc, fold change; HLA, human leukocyte antigen; HPLC, high-performance liquid chromatography; LC–MS/MS, liquid chromatography-coupled tandem mass spectrometry; mRNA, messenger ribonucleic acid

Additionally, immunopeptidomics was used as a peptide-agnostic approach to detect presentation of CVGBM-derived epitopes on HLA class I molecules of CVGBM mRNA-transfected HEK293T and THP-1 cells (Supplementary Table 2). Both cell lines express endogenous HLA-A*02:01 besides other class I alleles (Supplementary Table 1). Four of the six HLA-A*02:01-restricted epitopes encoded by CVGBM were presented by HLA class I molecules (Fig. 4, Supplementary Table 3, Supplementary data). No additional epitopes derived from other parts of the fusion protein, such as the epitope junctions, were detected. The specificity of the assay was corroborated by the finding that 98% of all detected peptides have been previously experimentally validated and are reported in the Immune Epitope Database [31] (Supplementary data). In contrast with HEK293T cells, THP-1 cells also express considerable amounts of HLA class II molecules. Therefore, an additional pull-down by anti-HLA class II antibodies was performed for the THP-1 cells; however, no CVGBM-derived HLA class II epitopes could be detected (Supplementary Table 2).

### In vivo functionality of CVGBM in mice

**Fig. 5:**
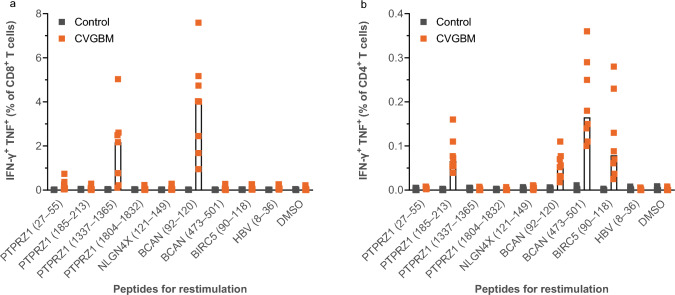
CVGBM induces CD8^+^ (**a**) and CD4^+^ (**b**) T-cell responses in mice. Naïve CB6F1 mice were injected intramuscularly with 5 µg CVGBM (orange) or control mRNA-LNP (black) on Days 0, 6 and 13. At Day 20, mice were sacrificed and splenocytes were isolated and restimulated with pooled peptides covering the individual antigen segments or DMSO. Cells were analysed by flow cytometry. The magnitude of the CD8^+^ (**a**) and CD4^+^ (**b**) T-cell responses against the antigen segments is presented as percentage of IFN-γ^+^ TNF^+^ cells of total CD4^+^ or CD8^+^ T-cell populations. Stimuli are ordered by position on fusion protein. Median values are plotted. Data are representative for two independent experiments. BCAN, brevican core protein; BIRC5, baculoviral IAP repeat-containing protein 5; DMSO, dimethyl sulfoxide; HBV, hepatitis B virus; IAP, inhibitor of apoptosis; IFN-γ, interferon gamma; LNP, lipid nanoparticle; mRNA, messenger ribonucleic acid; NLGN4X, neuroligin-4; PTPRZ1, receptor-type tyrosine-protein phosphatase zeta; TNF, tumour necrosis factor

To evaluate functionality of the CVGBM clinical candidate regarding protein processing and epitope presentation under physiological conditions, the induction of epitope-specific immune responses by CVGBM was analysed in CB6F1 mice. These hybrid mice express MHC molecules of both C57BL/6 and BALB/c strains and can therefore present a broader spectrum of epitopes on MHC molecules than the individual strains. Results confirmed the functionality of the multi-epitope CVGBM in vivo by inducing CD8^+^ and CD4^+^ T-cell responses against two and four of the nine segments, respectively (Fig. 5).

### In vivo immunogenicity and anti-tumoural efficacy of a murine surrogate immunotherapeutic

The anti-tumoural efficacy of CVGBM could not be tested in immunocompetent mouse models as they do not allow human GBM xenografts. Therefore, the mRNA-based B16 immunotherapeutic was designed as a murine surrogate, encoding a fusion protein containing ten epitopes of the murine B16.F10 melanoma tumour model (Supplementary Fig. 4; Supplementary Table 4). The B16 fusion protein had a similar design as CVGBM: epitopes were included as antigen segments of 29 aa separated by G_4_S linkers and the fusion protein was flanked by the same CTLA-4 domains. Additionally, the B16 fusion protein contained the synthetic T-helper epitope PADRE [32], but not an E5 degron.

**Fig. 6:**
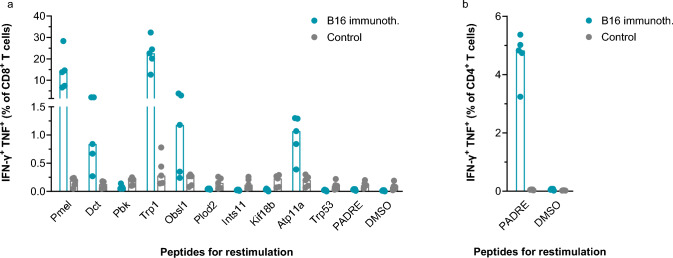
B16 immunotherapeutic induces CD8^+^ (**a**) and CD4^+^ (**b**) T-cell responses in mice. Naïve C57BL/6 mice were injected with 5 μg B16 immunotherapeutic (blue) or control mRNA-LNP (grey) on Day 0, 7 and 14. On Day 21, mice were sacrificed and splenocytes were isolated. Splenocytes were restimulated individually with the indicated peptides (29 aa each, 13 aa for PADRE) or DMSO as control. Cells were analysed by flow cytometry. The magnitude of the CD8^+^ and CD4^+^ T-cell responses against the segments was defined as percentage of IFN-γ^+^ TNF^+^ cells of the total CD8^+^ or CD4^+^ T-cell populations. Stimuli are ordered by position on fusion protein. Median values are plotted, and negative responses are omitted in (**b**). Data are representative for three independent experiments. aa, amino acids; CTLA-4, cytotoxic T-lymphocyte associated protein 4; DMSO, dimethyl sulfoxide; IFN-γ, interferon gamma; immunoth, immunotherapeutic; mRNA, messenger ribonucleic acid; PADRE, pan HLA-DR binding epitope; TNF, tumour necrosis factor

**Fig. 7:**
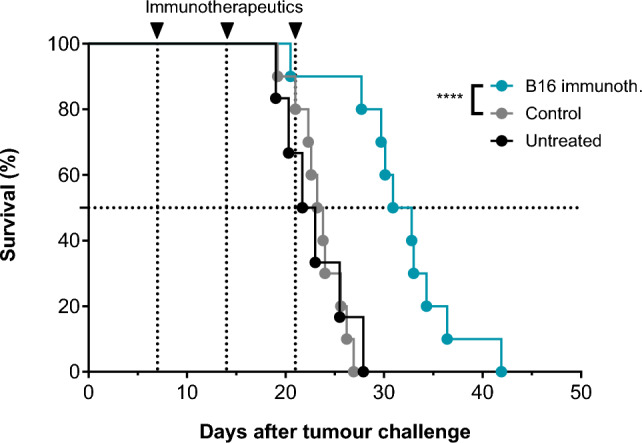
B16 immunotherapeutic demonstrates anti-tumoural efficacy in mice. Naïve C57BL/6 mice were challenged subcutaneously with 1 × 10^5^ B16.F10 cells in the lateral flank. Therapeutic treatment started when tumours were clearly palpable (Day 7; median tumour volume of ~ 11 mm^3^). At Day 7, 14 and 21 tumour-bearing mice were injected with 5 μg B16 immunotherapeutic (blue) or control mRNA-LNP (grey), or left untreated (black). Tumour size was determined twice per week using a calliper and tumour volume calculated. Survival of mice is plotted with the cut-off-volume for survival set at 1000 mm^3^. Significance was calculated using log-rank test (Mantel-Cox): ****p < 0.0001. immunoth, immunotherapeutic; mRNA, messenger ribonucleic acid

Treatment of naïve C57BL/6 mice with the B16 immunotherapeutic induced T-cell responses against six of the eleven encoded segments. CD8^+^ T-cell responses were observed against five segments and a CD4^+^ T-cell response against the T-helper epitope PADRE (Fig. 6). The immune response also translated into an anti-tumour effect and increased the median survival time of B16.F10 tumour-bearing C57BL/6 mice to 31.5 days following treatment with the B16 immunotherapeutic compared with 23.5 days and 22.4 days for control mRNA-LNP treated and untreated mice, respectively (Fig. 7). These results demonstrate the anti-tumoural potential of the protein and mRNA designs used for CVGBM.

## Discussion

Here, we describe the development and preclinical characterisation of CVGBM, an mRNA-based immunotherapeutic candidate for HLA-A*02:01-positive patients with surgically resected O^6^-methylguanine-DNA methyltransferase (MGMT)-unmethylated GBM following standard of care treatment. For this patient population, there is currently no efficacious treatment option available [4].

CVGBM was designed using CureVac’s proprietary RNActive® platform technology and comprises an mRNA sequence with unmodified nucleotides. All non-coding mRNA elements in the CVGBM mRNA were selected for optimal mRNA translation and are the same as in CV2CoV, CureVac’s second-generation SARS-CoV-2 mRNA vaccine [23]. The CVGBM mRNA is encapsulated in an LNP formulation that was previously tested in clinical trials of CVnCoV, CureVac’s first-generation SARS-CoV-2 mRNA vaccine, and is also utilised in the approved SARS-CoV-2 vaccine Comirnaty [33]. Due to their intrinsic immunostimulatory capacities, LNP-formulated mRNAs with unmodified nucleotides are a potent modality to deliver antigens for presentation on HLA class I and II molecules. They primarily induce CD8^+^ T-cell responses [22], which can exceed those induced by LNP-formulated mRNAs with modified nucleotides [34, 35], as well as CD4^+^ T-cell responses. In contrast, peptide-based immunotherapeutics often tend to induce primarily CD4^+^ T-cell responses [36]. Since cytoreductive therapy significantly impairs the immune system [37], the potential to induce strong CD8^+^ T-cell responses is particularly relevant for an antigen-based immunotherapeutic for GBM, where chemotherapy and radiotherapy remain the standard of care, and induced T cells must cross the blood–brain barrier to invade the immunologically “cold” tumour.

CVGBM encodes eight epitopes that are frequently presented on the surface of GBM tumour samples and have induced immune responses in previous clinical trials as peptide-based immunotherapeutics [14, 15]. The epitopes are derived from four TAAs that are either over-expressed (BCAN, BIRC5, PTPRZ1 [Supplementary Fig. 1b]) or where the epitope is over-presented (NLGNX4) in a large proportion of GBM tumours compared with most healthy tissues [28]. The use of TAA-derived epitopes has the advantage of allowing development of an off-the-shelf product for a tumour indication with low mutational burden such as GBM, where patients’ tumours express only few tumour-specific antigens [38]. However, this approach limits the therapy to patients carrying the HLA alleles to which the epitopes are restricted. To maximise the eligible patient population, the epitopes selected for CVGBM were either restricted to HLA-A*02:01 (BCAN, PTPRZ1, NLGNX4) as the most frequent MHC class I allele in the Caucasian population (up to 50% of individuals bearing this allele according to the allele frequency net database [39]), or to various HLA-DR alleles (BIRC5). As a result of the frequent overexpression of the selected TAAs and the restriction to HLA-A*02:01-positive patients, a high percentage of eligible GBM patients will have tumours presenting CVGBM-encoded epitopes of multiple TAAs, which might increase efficacy of the immunotherapeutic and reduce the risk of immune evasion by antigen loss. However, immune evasion can also occur by loss of the required HLA allele or defects in the HLA presentation pathway, although this is less common in GBM than other tumour types [40].

The epitopes encoded by CVGBM are concatenated into a single fusion protein (CVGBM core protein). This core protein is flanked by the SP and the transmembrane and cytoplasmic domains of CTLA-4, and a C-terminal E5 degron to optimise presentation of the CVGBM-encoded epitopes. Of note, the extracellular domain of CTLA-4, which acts as an immune checkpoint regulator [41], is not included in the fusion protein. The protein design for CVGBM was selected based on the comparison of several fusion protein variants that included the CVGBM core protein, two reporter epitopes, and different flanking regions that localised the variants to different subcellular compartments. In this comparison in murine cells, the protein design containing the CTLA-4 domains and the E5 degron demonstrated the best presentation of the reporter epitopes on MHC class I and II molecules. Immunopeptidomic analysis of two human cell lines transfected with the final CVGBM mRNA confirmed translation and processing of the CVGBM fusion protein and presentation of four of the six HLA-A*02:01-restricted epitopes on human cells; however, no CVGBM-derived HLA class II epitopes were detected, likely due to low expression of HLA class II molecules on THP-1 cells or the overall lower number of recovered HLA class II than HLA class I epitopes (Supplementary Table 2). Of note, CD4^+^ T-cell responses were detected in mice treated with CVGBM, demonstrating the ability for MHC class II presentation.

CTLA-4 is a membrane protein that is targeted by its SP via the ER to the cell surface; from here it is rapidly internalised to endosomes and lysosomal vesicles due to the cytoplasmic domain [42]. Two mechanisms could therefore contribute to the observed increased presentation of reporter epitopes derived from GBMr fusion proteins with CTLA-4 domains. The first is the targeting of the misfolded fusion protein comprising short antigen segments to the ER, where it might trigger the unfolded protein response and the ER-associated protein degradation pathway [43], generating more peptides for MHC class I loading. The second putative mechanism is the rapid internalisation of the fusion protein from the cell surface to the lysosomal compartment [42], which might provide more peptides for MHC class II loading in the lysosome. Additionally, MHC class I loading might be enhanced by the cytosolic, C-terminal E5 degron, which has been described to increase protein turnover and proteasomal degradation in the cytosol [30].

Physiologically, CTLA-4 is an immune checkpoint receptor expressed on Foxp3^+^ regulatory T cells and conventional T cells. CTLA-4 has also been detected in natural killer cells, B cells, dendritic cells and myeloid cells [44]. Consequently, there is a risk that the CTLA-4 domains in the CVGBM fusion protein could induce unwanted on-target, off-tumour CTLA-4-specific T-cell responses. Indeed, one peptide of the CTLA-4 transmembrane domain has been described as present on thymic tissue [45]. However, the immunopeptidomic analysis of the two human cell lines transfected with CVGBM mRNA did not detect any presentation of CTLA-4-derived epitopes on HLA molecules.

Finally, in vivo functionality of CVGBM was confirmed in mice, with epitope-specific CD8^+^ and CD4^+^ T-cell responses induced. While these responses were directed against epitopes presented on mouse MHCs that differ from the epitopes presented on human HLAs, they confirm the accurate translation of CVGBM mRNA into the encoded fusion protein and presentation of epitopes from different parts of the fusion protein on MHC class I and II molecules.

The anti-tumoural efficacy of CVGBM could not be tested with human GBM xenografts in animal models because xenografts are only possible in immunodeficient animals, while CVGBM requires immunocompetent animals for its mode-of-action. Recently, a murine model system was developed that circumvents this limitation by using an HLA-A*02:01-transgenic murine GBM tumour cell line, which can be transfected with human TAAs and implanted intracranially into immunocompetent HLA-A*02:01-transgenic mice [46]. For the present study, however, the syngeneic murine B16.F10 melanoma model was used instead to test the anti-tumoural potential of the mRNA and protein designed used for CVGBM. For this study, a surrogate mRNA-based immunotherapeutic was generated, encoding epitopes derived from the B16.F10 cell line and using the same mRNA design and a similar protein design as CVGBM. This B16 immunotherapeutic induced CD8^+^ and CD4^+^ T-cell responses in naïve mice from different parts of the fusion protein, including the N-terminal first (Pmel) and C-terminal last segment (PADRE). Consistently, our surrogate immunotherapeutic exhibited anti-tumoural efficacy in B16.F10 tumour-bearing mice, thus supporting the functionality of CVGBM mRNA and protein design.

Based on the findings reported here, the safety and immunogenicity of CVGBM are evaluated in a first-in-human Phase I trial (NCT05938387) in HLA-A*02:01-positive patients with newly diagnosed MGMT-unmethylated GBM (CNS WHO Grade 4) who had surgical resection and completed radiotherapy with or without concomitant temozolomide. Interim results from the initial dose escalation part of the study demonstrated that CVGBM was well tolerated with an acceptable safety profile and TAA-specific T-cell responses were induced in the majority of patients [47]. Further safety and immunogenicity results will be provided from the ongoing dose expansion part of the study.

## Supplementary Information

Below is the link to the electronic supplementary material.

Supplementary file1 (DOCX 906 KB)

Supplementary file2 (XLSX 352 KB)

## Data Availability

Relevant data that support these findings will be made available to qualified researchers upon reasonable request to the corresponding author.

## Abbreviations

BCAN: Brevican core protein
BIRC5: Baculoviral inhibitor of apoptosis repeat-containing protein 5
LNP: Lipid nanoparticle
NLGN4X: Neuroligin-4
PTPRZ1: Receptor-type tyrosine-protein phosphatase zeta
TAA: Tumour-associated antigen
TMZ: Temozolomide
