# Supplementary material for: Preclinical development of an mRNA-based multiepitope immunotherapeutic for glioblastoma
Source: Cancer Immunol Immunother. 2025 Oct 6;74(11):329. doi: 10.1007/s00262-025-04178-x (PMC12500496; doi:10.1007/s00262-025-04178-x)
Supplement: Supplementary file 1 — Supplementary file1 (DOCX 906 KB) [file 262_2025_4178_MOESM1_ESM.docx]

**Supplementary materials**

**Supplementary Table 1. HLA alleles of HEK293T and THP-1 cell lines**

|  | **HEK293T [1]** | **THP-1 [2]** |
| --- | --- | --- |
| Class I | HLA-A A*02:01, 03:01  HLA-B B*07:02, 07:02/35:08  HLA-C C*07:02, 07:02 | HLA-A A*02:01:01, 24:02:01  HLA-B B*15:11:01, 35:01:01  HLA-C C*03:03:01, 03:03:01 |
| Class II | HLA-DQ DQA1*01:02, 01:02  DQB1*06:02, 06:02  HLA-DR DRB1*15:01, 15:01  HEK293T cells express only very few class II molecules | HLA-DP DPA1*01:03:01, 02:02:02  DPB1*02:01:02, 04:02:01  HLA-DQ DQB1*05:01:01, 06:02:01  HLA-DR DRA*01:01:01, 01:01:01  DRB1*01:01:01, 15:01:01 |

**Supplementary Table 2. Summary of epitopes detected by immunopeptidomics on mRNA-transfected HEK293T and THP-1 cells**

| **Sample** | **Unique class I epitopes (PSM)** | **CVGBM-derived class I epitopes (PSM)** | **Unique class II epitopes (PSM)** | **CVGBM-derived class II epitopes (PSM)** |
| --- | --- | --- | --- | --- |
| HEK293T transfected with control mRNA | 804 (7301) | 0 (0) | - | - |
| HEK293T transfected with CVGBM-mRNA | 715 (6669) | 4 (262) | - | - |
| THP-1 transfected with control mRNA | 1912 (19142) | 0 (0) | 181 (1585) | 0 (0) |
| THP-1 transfected with CVGBM-mRNA | 2168 (23351) | 3 (304) | 240 (2290) | 0 (0) |

HEK293T and THP-1 cells were transfected with CVGBM or control mRNA. Presented epitopes were purified separately using antibodies against HLA class I or II molecules and identified by LC/MS-MS. Data from five technical replicates were processed together and unique class I and II epitopes are listed. The number of identified peptide spectra matched (PSM) are given in brackets. Peptides were not filtered by predicted HLA binding.

HLA, human leukocyte antigen; LC/MS-MS, liquid chromatography-coupled tandem mass spectrometry; mRNA, messenger ribonucleic acid; PSM, peptide spectra matched

**Supplementary Table 3. CVGBM-derived epitopes detected by immunopeptidomics on CVGBM mRNA-transfected HEK293T and THP-1 cells**

| **Epitope** | **Name** | **Source protein** | **THP-1**  **detected in runs** | **HEK293T**  **detected in runs** |
| --- | --- | --- | --- | --- |
| AIIDGVESV | PTP-003 | Receptor-type tyrosine-protein phosphatase zeta (PTPRZ_HUMAN) | 5/5 | 5/5 |
| KVFAGIPTV | PTP-005 | Receptor-type tyrosine-protein phosphatase zeta (PTPRZ_HUMAN) | 5/5 | 5/5 |
| NLDTLMTYV | NLGN4X-001 | Neuroligin-4 (NLGNX_HUMAN) | 5/5 | 5/5 |
| FLPSDFFPSV | HBV-001 | Capsid protein (CAPSD_HBVA3); HBV | 0/5 | 5/5 |

Detailed information on the CVGBM-derived epitopes detected in the immunopeptidomics data from Supplementary Table 2.

HBV, Hepatitis B virus; mRNA, messenger ribonucleic acid; NLGN4X, neuroligin-4; PTPRZ1, receptor-type tyrosine-protein phosphatase zeta

**Supplementary Table 4. Epitopes encoded by surrogate murine mRNA-based B16 immunotherapeutic**

| **Epitope name** | **Amino acid position** | **Mutation** | **Epitope type** | **T-cell response**  **in naïve mice**  **after**  **vaccination**  **(literature)** | **Observed T-cell response**  **in naïve mice**  **after B16**  **immunotherapeutic** | **Reference** |
| --- | --- | --- | --- | --- | --- | --- |
| Pmel | 15–43 | S27P | TAA | CD8 | CD8 | [3] |
| Dct (TRP2) | 170–198 | - | TAA | CD8 | CD8 | [4, 5] |
| Pbk | 131–159 | V145D | Neoepitope | CD8 | - | [6, 7] |
| Trp1 | 445–473 | A463M | TAA | CD8 | CD8 | [8] |
| Obsl1 | 1750–1778 | T1764M | Neoepitope | CD8 | CD8 | [6, 7] |
| Plod2 | 516–544 | F530V | Neoepitope | CD4 | - | [6, 7] |
| Ints11 (Cpsf3l) | 300–328 | D314N | Neoepitope | CD4 | - | [6, 7] |
| Kif18b | 725–753 | K739N | Neoepitope | CD4 | - | [6, 7] |
| Atp11a | 508–536 | R522S | Neoepitope | CD4 | CD8 | [6, 7] |
| Trp53 (p53) | 220–248 | M234I | Neoepitope | CD8 | - | [9] |
| PADRE | - | - | Synthetic epitope | CD4 | CD4 | [10] |

mRNA, messenger ribonucleic acid; PADRE, pan HLA-DR binding epitope; TAA, tumour-associated antigen

**Supplementary Figure 1. Expression of selected TAAs in GBM tumours and healthy tissues**


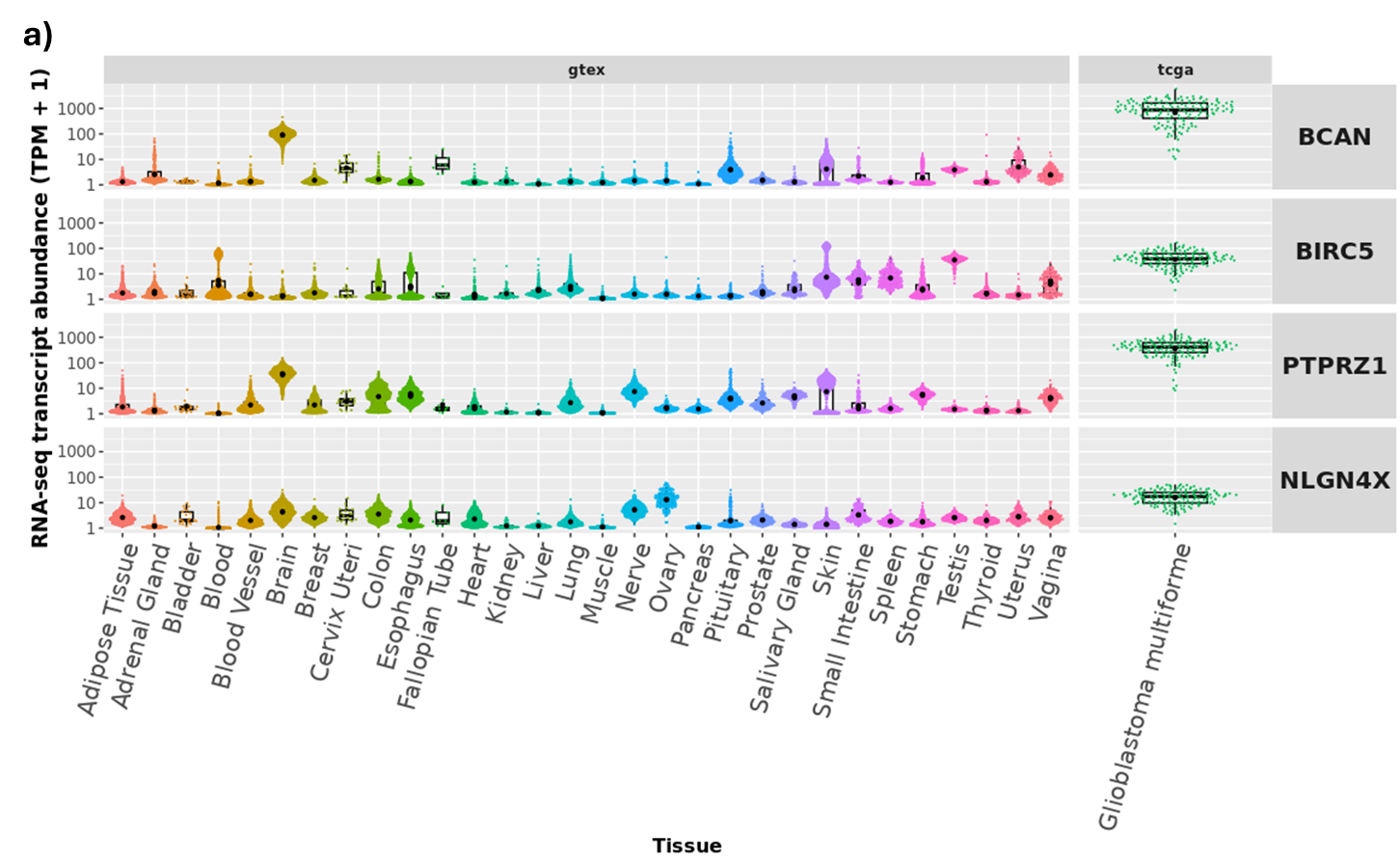


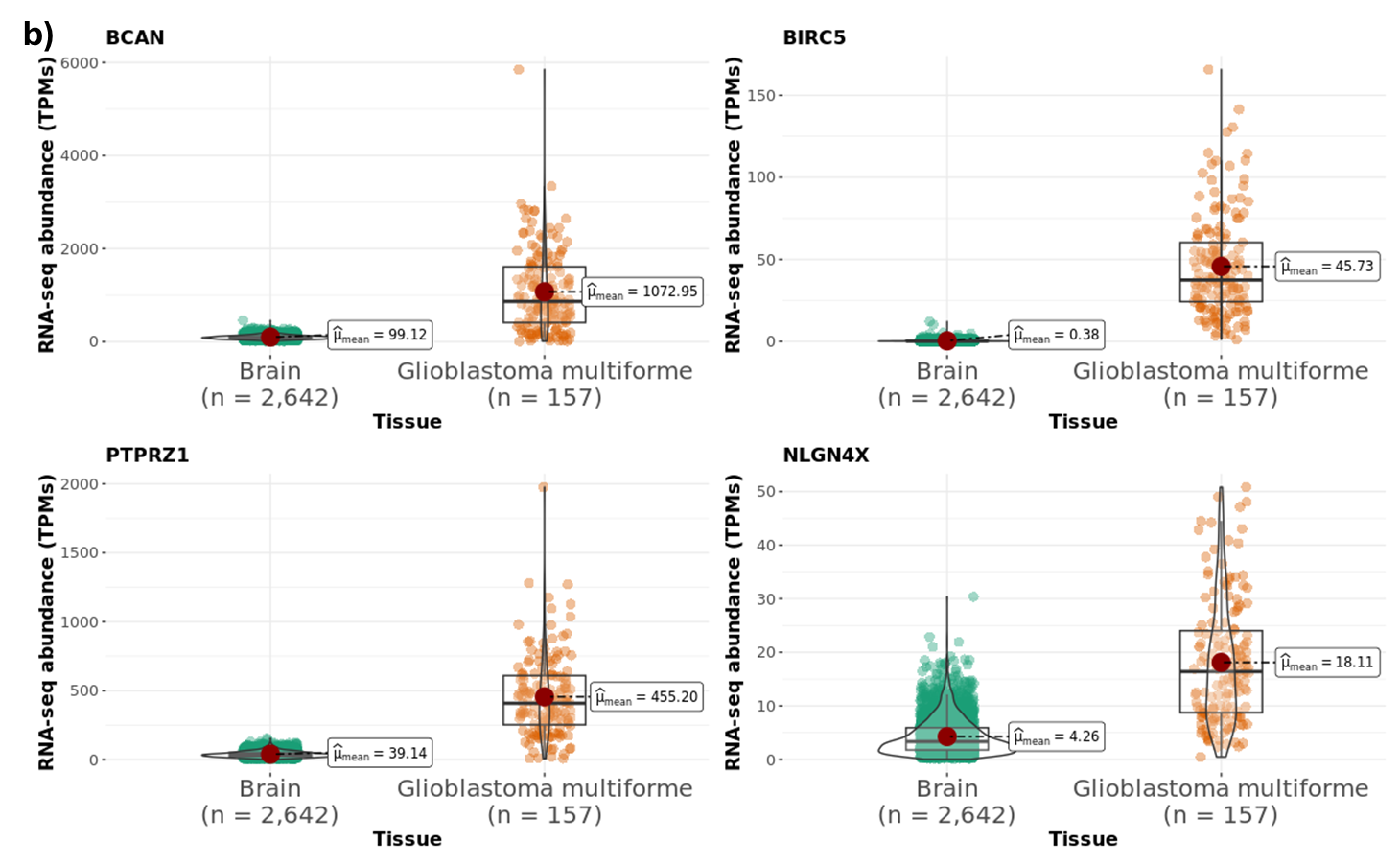


a) Comparison of expression in GBM tumour tissue versus healthy tissue

b) Detailed comparison of GBM tumour tissue versus healthy brain tissue. The mean expression is depicted in red points. BCAN, BIRC5, PTPRZ1 show the largest mean expression difference between GBM and brain tissue

The gene expression for all antigens in healthy and tumour tissues was analysed using the GTEx portal (V8) [11], and TCGA (v37.0) [12], respectively. The TCGA project “GBM” (Glioblastoma multiforme, containing RNA-seq data) was selected and subset to primary tumour tissue. To reduce batch effects and improve comparability, all raw RNA-seq expression data were internally processed employing kallisto (V0.48.0; a widely used tool to calculate transcript abundance) [13]. A total of 157 samples met the described criteria and were used for this analysis

BCAN, brevican core protein; BIRC5, baculoviral IAP repeat-containing protein 5; GBM, glioblastoma multiforme; GTEx, Genotype Tissue Expression; IAP, inhibitor of apoptosis; PTPRZ1, receptor-type tyrosine-protein phosphatase zeta; TCGA, The Cancer Genome Atlas; TPM, transcripts per million; TAA, tumour-associated antigen

**Supplementary Figure 2. Structural overview of the CVGBM mRNA and the encoded fusion protein**

**
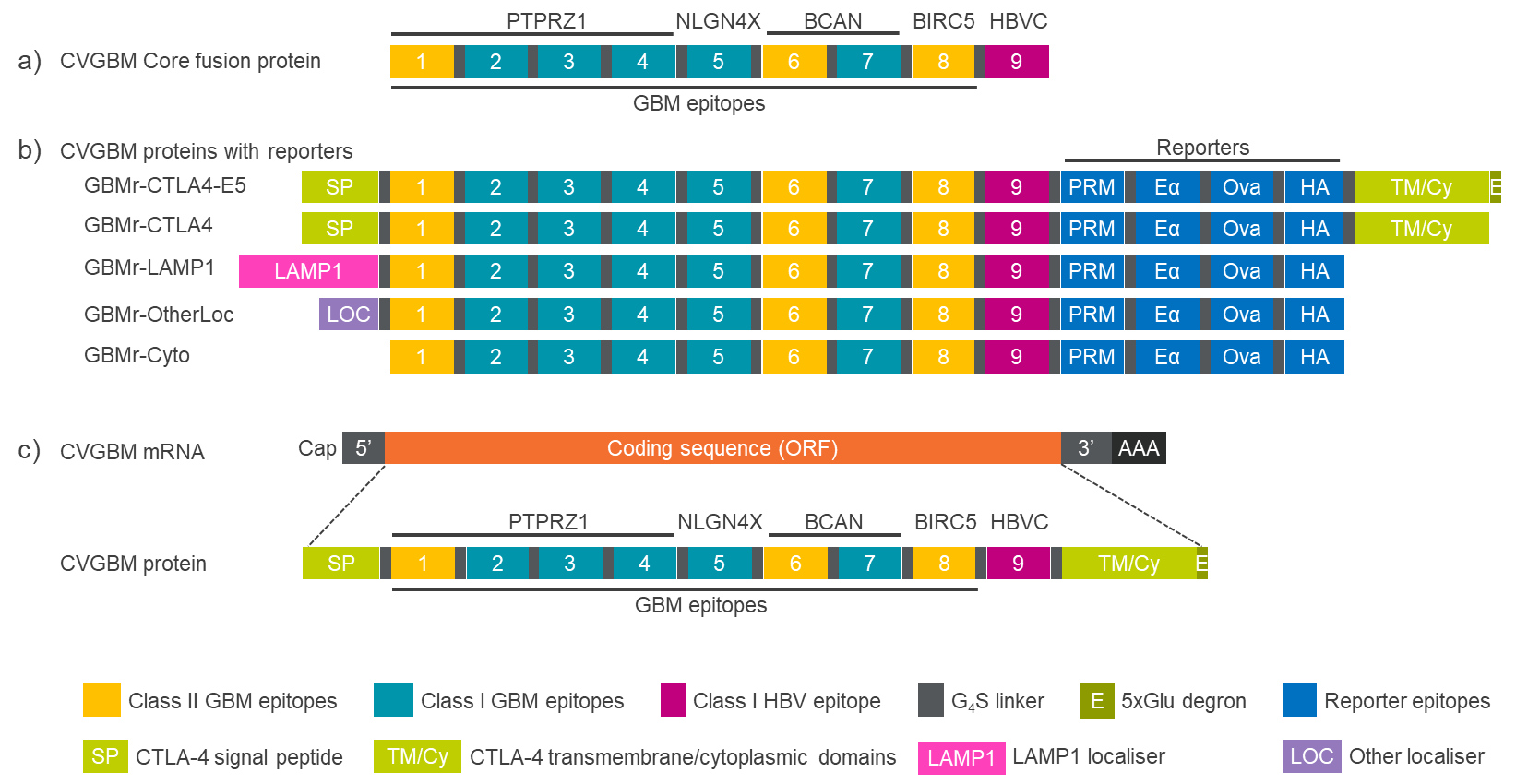
**

a) CVGBM core fusion protein consists of nine epitopes, each in an antigen segment of 29 aa each: 1: PTPRZ1 (aa 27–55; comprises PTP-010); 2: PTPRZ1 (aa 185–213; comprises PTP-003); 3: PTPRZ1 (aa 1337–1365; comprises PTP-005); 4: PTPRZ1 (aa 1804–1832; comprises PTP-013); 5: NLGN4X (aa 121–149; comprises NLGN4X-001); 6: BCAN (aa 92–120; comprises BCA-005); 7: BCAN (aa 473–501; comprises BCA-002); 8: BIRC5 (aa 90–118; comprises BIR-002); 9: HBV capsid protein (aa 8–36; comprises HBV-001), separated by linkers (G_4_S)

b) CVGBM fusion proteins with reporters (GBMr). The reporters comprise three segments of 29 aa each (H2-Eα [aa 71–99; comprises Eα], SERPINB14 [aa 247–275; comprises SIINFEKL, Ova] and PRAME [aa 290–318, PRM]) as well as a 3xHA-tag (31 aa). The fusion proteins contain different localisers that target them to different subcellular localisations. GBMr-CTLA4-E5 and GBMr-CTLA4 contain the SP of CTLA‑4 (aa 1–35, SP) and the TM/Cy domains of CTLA‑4 (aa 162–223, TM/Cy) to localise the protein to the ER and the endosomal pathway. GBMr-CTLA4-E5 contains a C-terminal E5 degron for enhanced degradation. GBMr-CTLA4-LAMP1 contains the SP and further aa sequences derived from LAMP1 to localise the fusion protein to the inside of lysosomes and late endosomes. GBMr-OtherLoc contains a different N-terminal LOC to localise the fusion protein to the ER. GBMr-Cyto lacks LOCs and is cytoplasmic

c) Schematic representations of the CVGBM mRNA and the encoded fusion protein

aa, amino acid residues; BCAN, brevican core protein; BIRC5, baculoviral IAP repeat-containing protein 5; CTLA-4, cytotoxic T-lymphocyte-associated protein 4; ER, endoplasmic reticulum; GBM, glioblastoma multiforme; HBV, hepatitis B virus; IAP, inhibitor of apoptosis; LAMP1, lysosomal-associated membrane protein 1; LOC, localisation domain; MHC, major histocompatibility complex; NLGN4X, neuroligin-4; ORF, open reading frame; Ova, chicken ovalbumin; PTPRZ1, receptor-type tyrosine-protein phosphatase zeta; SP, signal peptide; TM/Cy, transmembrane/cytoplasmic domains; UTR, untranslated region

**Supplementary Figure 3. Presentation kinetic of the MHC class II epitope Eα**


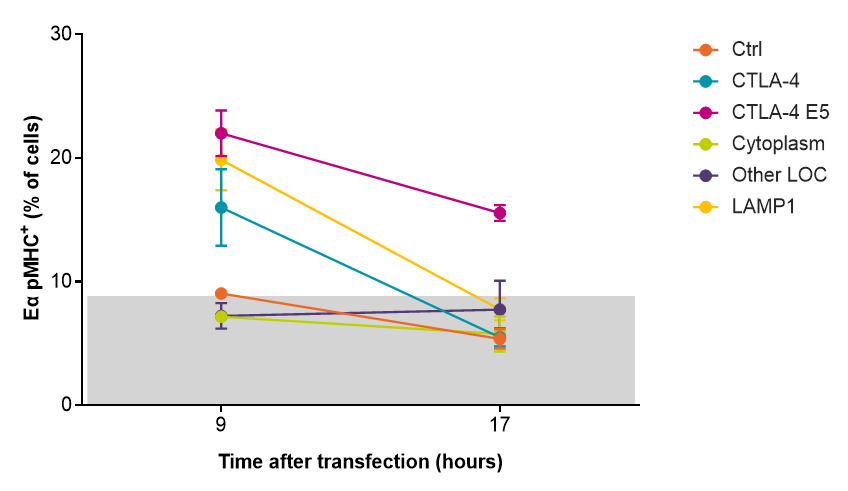


Presentation of Eα as a percentage of Eα pMHC^+^ cells detected by flow cytometry on fresh BMDCs derived from C57BL/6 mice 9 and 17 hours after transfection with the indicated mRNA constructs. The median values of two technical replicates are depicted. The background level of Eα pMHC staining (Ctrl at 9 hours) is shown in grey. Error bars represent standard deviation

BMDC, bone marrow dendritic cells; ctrl, control; CTLA-4, cytotoxic T-lymphocyte-associated protein 4; LAMP1, lysosomal-associated membrane protein 1; LOC, localisation domain; (p)MHC, (peptide) major histocompatibility complex

**Supplementary Figure 4. Structural overview of the B16 fusion protein**


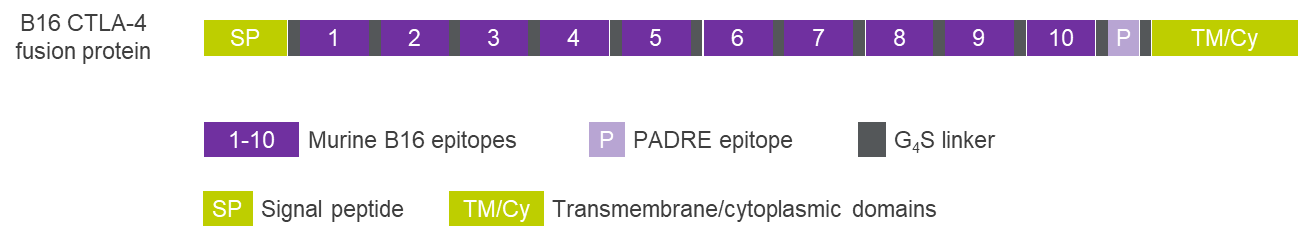


The B16 fusion protein consists of the SP of CTLA-4 (aa 1–35), 10 epitopes derived from the B16.F10 melanoma cell line in antigen segments of 29 aa each: 1: Pmel (15–43, S27P), 2: Dct (170–198), 3: Pbk (131–159, V145D), 4: Trp1(445–473, A463M), 5: Obsl1 (1750–1778, T1764M), 6: Plod2 (516–544, F530V), 7: Ints11 (300–328, D314N), 8: Kif18b (725–753, K739N), 9: Atp11a (508–536, R522S), 10: Trp53(220–248, M234I); PADRE (13 aa), and the TM/Cy domains of CTLA-4 (162–223), all separated by linkers (G_4_S)

CTLA4, cytotoxic T-lymphocyte-associated protein 4; PADRE, pan HLA-DR binding epitope; SP, signal peptide; TM/Cy, transmembrane/cytoplasmic domains

**References**

1. Boegel S, Löwer M, Bukur T, Sahin U, Castle JC (2014) A catalog of HLA type, HLA expression, and neo-epitope candidates in human cancer cell lines. Oncoimmunology. 3: e954893. doi: 10.4161/21624011.2014.954893

2. Cell line: THP-1. https://celldive.dsmz.de/celllines/THP-1. Accessed 15 Apr 2025

3. van Stipdonk MJ, Badia-Martinez D, Sluijter M, Offringa R, van Hall T, Achour A (2009) Design of agonistic altered peptides for the robust induction of CTL directed towards H-2Db in complex with the melanoma-associated epitope gp100. Cancer Res. 69: 7784-92. doi: 10.1158/0008-5472.Can-09-1724

4. Bloom MB, Perry-Lalley D, Robbins PF, Li Y, el-Gamil M, Rosenberg SA, Yang JC (1997) Identification of tyrosinase-related protein 2 as a tumor rejection antigen for the B16 melanoma. J Exp Med. 185: 453-9. doi: 10.1084/jem.185.3.453

5. Schreurs MW, Eggert AA, de Boer AJ, Vissers JL, van Hall T, Offringa R, Figdor CG, Adema GJ (2000) Dendritic cells break tolerance and induce protective immunity against a melanocyte differentiation antigen in an autologous melanoma model. Cancer Res. 60: 6995-7001.

6. Castle JC, Kreiter S, Diekmann J et al. (2012) Exploiting the mutanome for tumor vaccination. Cancer Res. 72: 1081-91. doi: 10.1158/0008-5472.Can-11-3722

7. Kreiter S, Vormehr M, van de Roemer N et al. (2015) Mutant MHC class II epitopes drive therapeutic immune responses to cancer. Nature. 520: 692-6. doi: 10.1038/nature14426

8. Guevara-Patiño JA, Engelhorn ME, Turk MJ et al. (2006) Optimization of a self antigen for presentation of multiple epitopes in cancer immunity. J Clin Invest. 116: 1382-90. doi: 10.1172/jci25591

9. Mansour M, Pohajdak B, Kast WM, Fuentes-Ortega A, Korets-Smith E, Weir GM, Brown RG, Daftarian P (2007) Therapy of established B16-F10 melanoma tumors by a single vaccination of CTL/T helper peptides in VacciMax. J Transl Med. 5: 20. doi: 10.1186/1479-5876-5-20

10. Alexander J, Sidney J, Southwood S et al. (1994) Development of high potency universal DR-restricted helper epitopes by modification of high affinity DR-blocking peptides. Immunity. 1: 751-61. doi: 10.1016/s1074-7613(94)80017-0

11. Lonsdale J, Thomas J, Salvatore M et al. (2013) The Genotype-Tissue Expression (GTEx) project. Nature Genetics. 45: 580-5. doi: 10.1038/ng.2653

12. National Cancer Institute Center for Cancer Genomics (2024) The Cancer Genome Atlas Program (TCGA). https://www.cancer.gov/ccg/research/genome-sequencing/tcga. Accessed October 2024

13. Bray NL, Pimentel H, Melsted P, Pachter L (2016) Near-optimal probabilistic RNA-seq quantification. Nature Biotechnology. 34: 525-7. doi: 10.1038/nbt.3519
